# Quantification of muscles activations and joints range of motions during oil palm fresh fruit bunch harvesting and loose fruit collection

**DOI:** 10.1038/s41598-021-94268-4

**Published:** 2021-07-22

**Authors:** Yu Xuan Teo, Yon Sin Chan, Darwin Gouwanda, Alpha Agape Gopalai, Surya Girinatha Nurzaman, Subbiah Thannirmalai

**Affiliations:** 1 School of Engineering, Monash University Malaysia, Jalan Lagoon Selatan, Bandar Sunway, 47500 Subang Jaya, Selangor Malaysia; 2 Sime Darby Research, Jalan Pulau Carey, 42960, Pulau Carey, Selangor Malaysia

**Keywords:** Biomechanics, Biomedical engineering, Electrical and electronic engineering, Mechanical engineering, Health occupations

## Abstract

Although global demand for palm oil has been increasing, most activities in the oil palm plantations still rely heavily on manual labour, which includes fresh fruit bunch (FFB) harvesting and loose fruit (LF) collection. As a result, harvesters and/or collectors face ergonomic risks resulting in musculoskeletal disorder (MSD) due to awkward, extreme and repetitive posture during their daily work routines. Traditionally, indirect approaches were adopted to assess these risks using a survey or manual visual observations. In this study, a direct measurement approach was performed using Inertial Measurement Units, and surface Electromyography sensors. The instruments were attached to different body parts of the plantation workers to quantify their muscle activities and assess the ergonomics risks during FFB harvesting and LF collection. The results revealed that the workers generally displayed poor and discomfort posture in both activities. Biceps, multifidus and longissimus muscles were found to be heavily used during FFB harvesting. Longissimus, iliocostalis, and multifidus muscles were the most used muscles during LF collection. These findings can be beneficial in the design of various assistive tools which could improve workers' posture, reduce the risk of injury and MSD, and potentially improve their overall productivity and quality of life.

Oil palm trees are the most efficient oil-bearing crop in the world, requiring only 0.26 hectares of land to produce 1 tonne of oil while soybean, sunflower and rapeseed require 2.22, 2.0 and 1.52 hectares, respectively, to have the same amount of oil^1^. Its efficiency makes oil palm plantations one of the most profitable forms of land use in the tropics. As a result, the palm oil industry is viewed as a significant contributor to the national economy, assisting and driving rapid economic growth while contributing to the alleviation of rural poverty.

Despite its economic potential, harvesting of oil palm presents several challenges as a result of the tree's natural build-up. Oil palms are single-stemmed and can grow well over 20 m tall. They also have heavy leaf foliage (frond)^2^, which acts to protect the oil palm flower from developing into a large fruit cluster/bunch. The fruit bunch, commonly known as Fresh Fruit Bunch (FFB), is located at the top of the tree and can weigh between 10 and 25 kg with 1000 to 3000 fruitlets per bunch^3^. These factors (tree height and fronds) complicate the harvesting process, causing it to be heavily reliant on the human workforce to execute an effective harvesting routine/yield.

Labour-intensive activities in the upstream production of palm oil typically include FFB harvesting and loose fruit (LF) collection as primary activities^4^. During the FFB harvesting, the harvester needs to visually identify and cut ripe FFB, as shown in Fig. 1A and B. However, this task is further complicated with the presence of fronds limiting the harvester's access to the FFB^2^. Therefore, the harvester needs to first carefully prune the fronds (to not damage the tree and its future yield) before harvesting the identified FFB^5^. Harvesting of FFB will cause the dislodgement of fruitlets from the bunch, upon its impact with the ground. These LF form the second activity, which is the LF collection. The LF collectors are often in a crouched position to ease the raking/sweeping of the LF into a collector bin as shown in Fig. 1C and D. These two tasks (FFB harvesting and LF collection) physically strain the musculoskeletal system and pose a high risk of musculoskeletal disorder (MSD) especially considering that the workers are repeatedly^6^ in such positions throughout the day to execute their tasks, as their wages depend on their yield for the day^7^.

**Figure 1:**
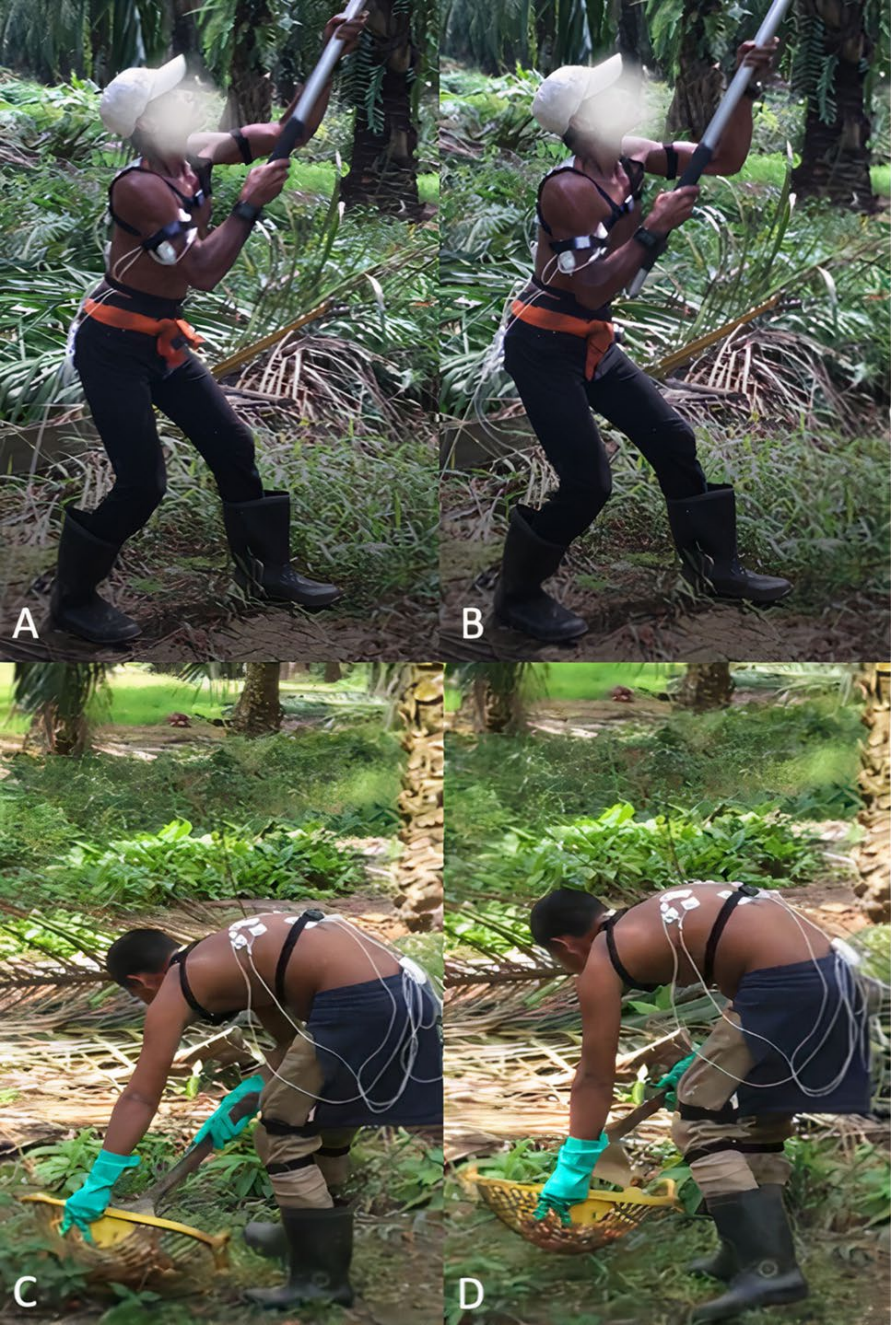
(**A**,**B**): An example of an oil palm FFB harvesting posture (**C**,**D**): An example of an oil palm LF collection posture.

Indirect approaches were adopted in the previous studies to investigate the risk and susceptibility of MSD among oil palm plantation workers. These methods involved the use of survey^2, 7^ and the analysis of the visual observation using Ovako Working Posture Analysis System (OWAS)^2, 5^, Rapid Entire Body Assessment (REBA)^8^ and Rapid Upper Limb Assessment (RULA)^9, 10^. They also show that the workers do suffer from discomfort and pain in various parts of the body. However, none of these studies investigated the dynamic behaviours of the workers by using direct measurements. In this regard, rapid advances in micro-miniaturization of sensors, microcontrollers, and wireless technology have enabled motion capture and analysis outside the laboratory environment. For example, wearable technologies such as a wearable module for recording worker position^11^ were applied in orchards and plantations to understand the workers' working pattern and increase their productivity. One of the popular technologies is the wireless Inertial Measurement Units (IMUs). IMUs can be used in an outdoor setting to measure the joint angles of various body parts, and data can be wirelessly transmitted to the nearby workstation, such as laptop, mobile phone, or tablet^12, 13^. Studies also reported their accuracy and reliability, which are comparable to the conventional optical motion capture system^14, 15^.

Complementing the IMUs with wireless surface electromyography (sEMG) can provide a comprehensive outlook of the workers' behaviour when they perform their daily routines. It enables the identification of the muscles that workers heavily rely on and consequently allows the assessment of the ergonomic risk of MSD in their workplaces. The combination of IMUs and sEMG has been widely used in many industrial and agricultural applications, including banana harvesting^16^, construction roadwork^17^ and manual planting^18^. Hence, in this study, we seek to quantitatively assess the dynamic behaviour of the workers during FFB harvesting and LF collection using wireless IMUs and sEMG and discuss the likelihood of MSD due to the behavior. The outcome of this study is expected to serve as the fundamental to further improve the posture of the oil palm workers, design better harvesting tools, and design assistive devices such as advanced exoskeleton suits that can alleviate the workers' daily load and improve their overall quality of life.

The rest of the paper is organized as follows. In “Methodology” section, the methodology is explained, and results and analysis will be presented in the subsequent chapter. In “Discussions” section, discussions were made on the processed data. “Conclusion” section concludes the critical points and further improvements to the project.

## Methodology

### Sensors

Two types of measurements were used in this study—EMG sensors and IMU sensors. EMG measures the electrical activation associated with muscular contraction (mV)^6^, either by inserting the electrodes into the muscle or by applying them on the surface of the skin^18^. The latter, known as surface electromyography (sEMG), was selected for our study due to its non-invasive characteristics^19, 20^. This work uses a wireless sEMG unit (Biosignalplux, Lisbon, Portugal) that has 7 data acquisition channels and one data synchronization channel. It has a sampling rate of 1000 Hz and able to transmit data wirelessly within a radius of 10 m^21^. The EMG electrodes were placed based on SENIAM convention^22, 23^. Cram's convention^24^ was used for muscles that SENIAM did not cover.

IMU is a motion sensor that measures kinematics such as linear acceleration, angular velocity and orientation of an object^25^. When several IMUs are used together and placed in a specific configuration on the human body, it is able to estimate the joint angle. It has been widely used in various research related to gait, sports, rehabilitative exercise and any kinematic tracking outside of the laboratory due to its miniature size and wireless data acquisition capabilities^25, 26^. A total of six wireless IMUs (OPAL, by APDM, Portland, OR, USA) with accelerometer range of ± 6 g, magnetometer range of ± 6 Gauss, X and Y axis gyroscope range of ± 2000°/sec and Z axis gyroscope range of ± 1500°/sec were used in this study and placed at left and right upper arm, left and right forearm, sternum and lumbar to estimate the joint angle. The sampling rate was set at 128 Hz. Both IMU and sEMG data collection were synchronized using an external trigger.

### Muscles selection

A preliminary study was first conducted to identify the primary muscles involved in the FFB harvesting and LF collection. This part of the study includes manual visual observation on video recordings of FFB harvesting and LF collection, recorded in a local Malaysia oil palm plantation. Based on the videos, we identified 14 primary motions during FFB harvesting involving the motions of the upper extremity, including the back, shoulder and elbow. We also identified four primary motions during LF collection involving the motions of the back. The primary muscles which are responsible for these motions are tabulated in Table 1. Only superficial muscles were selected^27^, as measurements would be carried out using sEMG electrodes^19, 28, 29^. However, although rectus abdominis, internal oblique and external oblique are superficial muscles, they were not shortlisted because these muscles are usually covered by a thick layer of adipose tissue which can attenuate the sEMG signal^24^.

**Table 1:** Fundamental motions and muscles involved during FFB harvesting and LF collection^30, 31^.

FFB harvesting		LF collection	
Motion	Muscles	Motion	Muscles
Back extension	Spinalis Longissimus Iliocostalis Multifidus	Back extension	Spinalis Longissimus Iliocostalis Multifidus
Back flexion	Rectus abdominis	Back flexion	Rectus abdominis
Back lateral bending	Longissimus Iliocostalis Multifidus Internal oblique External oblique	Back lateral bending	Longissimus Iliocostalis Multifidus Internal oblique External oblique
Back rotation	Longissimus Iliocostalis Multifidus Internal oblique External oblique	Back rotation	Longissimus Iliocostalis Multifidus Internal oblique External oblique
Scapular elevation	Upper trapezius Levator scapulae		
Scapular rotation	Upper trapezius Middle trapezius Rhomboids		
Scapular retraction	Upper trapezius Middle trapezius Lower trapezius		
Elbow flexion	Biceps		
Elbow supination	Biceps Supinator		
Elbow extension	Triceps		
Shoulder flexion	Clavicular head of the pectoralis major Sternocostal head of the pectoralis major Anterior deltoid Biceps		
Shoulder extension	Triceps Latissimus dorsi Teres major		
Shoulder abduction	Deltoid Biceps		
Shoulder adduction	Triceps Clavicular head of the pectoralis major Sternocostal head of the pectoralis major Latissimus dorsi		

Since the sEMG unit only allows for the measurement of seven muscles simultaneously, a preliminary experiment was conducted to identify the muscles that have the highest activation level in both activities:

*FFB harvesting* Three experienced right-handed male FFB harvesters were recruited and requested to perform the harvesting activity for 30 s, this activity was repeated six times, with a 30 s rest between trials. There are only 12 superficial muscles suitable for sEMG testing and in this experiment, all of them were investigated: clavicular head of the pectoralis major, sternocostal head of pectoralis major, anterior deltoid, latissimus dorsi, upper trapezius, middle trapezius, lower trapezius, biceps, triceps, longissimus, iliocostalis and multifidus.

*LF collection* Three experienced right-handed male LF collectors were recruited to perform the LF collection activity for 30 s, this activity was repeated six times with a 30 s rest between trials. There are only three superficial muscles suitable for sEMG testing and in this experiment, all of them were investigated: longissimus, iliocostalis and multifidus.

This study indicated that the upper trapezius, middle trapezius, lower trapezius, triceps, biceps, longissimus and multifidus muscles had the highest muscle activation during FFB harvesting. It also showed that the longissimus, iliocostalis, and multifidus muscles had significant muscle activation during LF collection.

### Experiment setup

Two subject groups were recruited for this study, representative of the FFB harvester and LF collector. Both groups consisted of males with at least five years of working experience in their respective harvesting and collecting activity.

**Figure 2:**
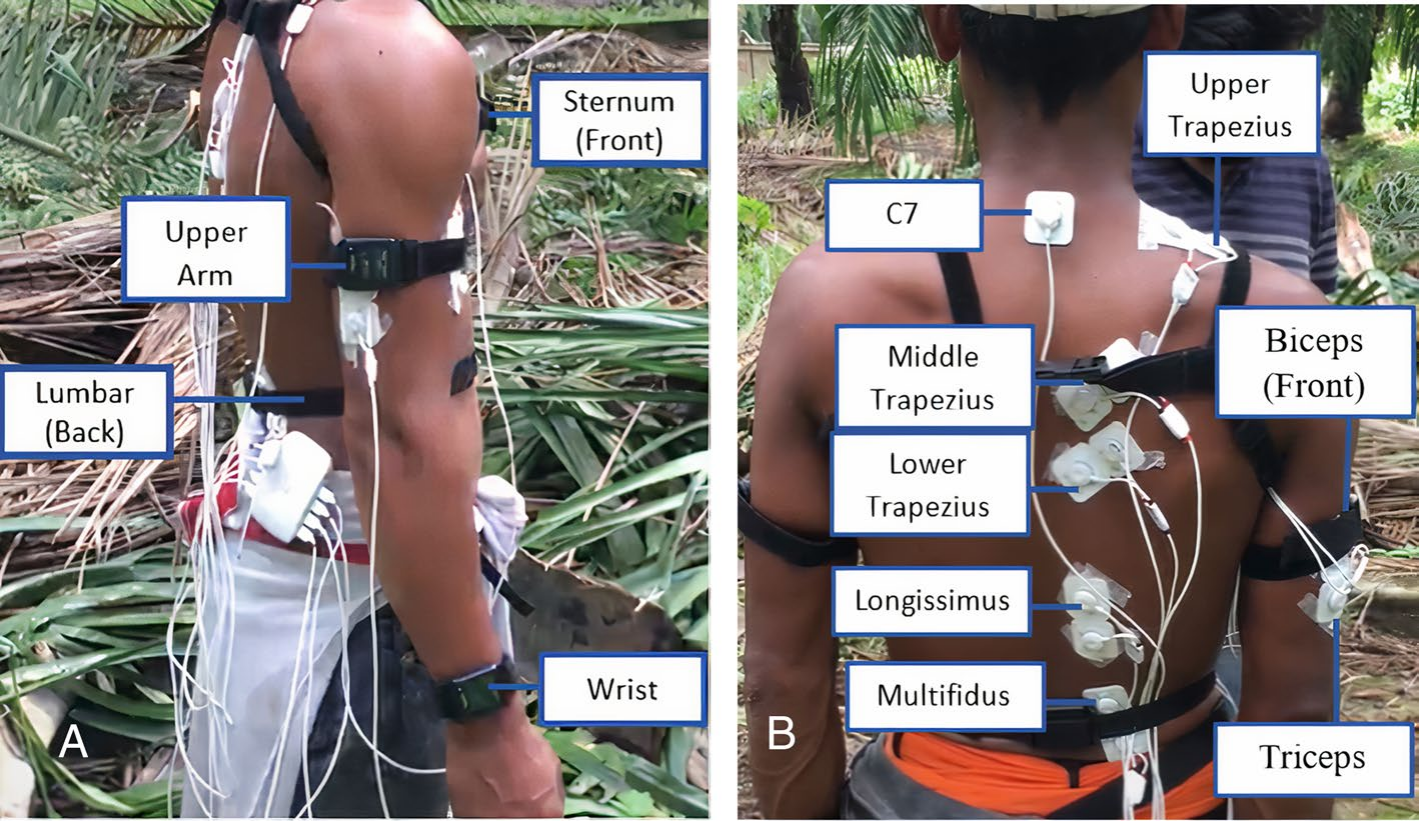
IMU Sensor placement (**A**) and EMG Sensor placement (**B**) on Harvester's body.

For FFB harvesting: Eight right-handed harvesters were recruited (n = 8; Age: 33.5 ± 5.9 years; Height: 167.3 ± 5.1 cm; Weight: 56.6 ± 3.7 kg). For better consistency and comparability of the result, FFB harvesters were advised to harvest trees that were approximately 3 m and 5 m in height^32^. Six IMUs were used to measure the joint angles of the upper extremity and torso movement. The IMUs were securely placed on sternum, lumbar, upper arms and wrists using lock and strap fasteners, as shown in Fig. 2A. EMG electrodes were also placed on the harvester's body to measure the activations of the upper trapezius, middle trapezius, lower trapezius, triceps, biceps, longissimus and multifidus muscles, as shown in Fig. 2B, together with the reference electrode at C7 region.

**Figure 3:**
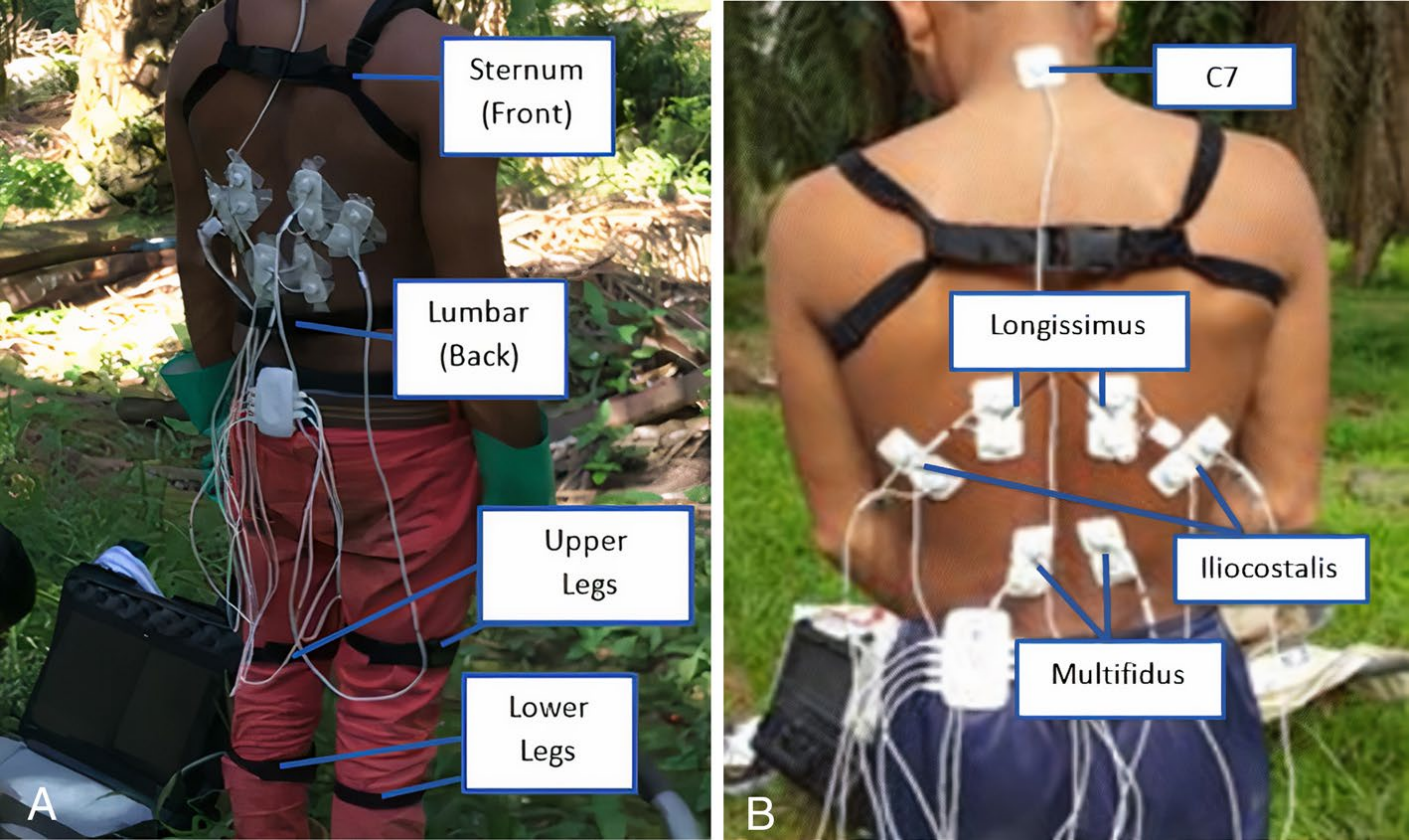
IMU Sensor placement (**A**) and EMG sensor placement (**B**) on Collector's Body.

For LF collection: Eight right-handed collectors were recruited (n = 8; Age: 30.8 ± 2.9 years; Height: 167 ± 6.5 cm; Weight: 63.1 ± 6.8 kg). Six IMUs were again used to measure the joint angles of the lower extremity and torso movements. They were placed on the subject's sternum, lumbar, upper legs and lower legs and secured using lock and strap fasteners as shown in Fig. 3A to minimize skin and soft tissue artefact, as shown in Fig. 3A. EMG electrodes were placed on the collector's body as shown in Fig. 3B to measure the activations of the longissimus, multifidus and iliocostalis muscles.

Both groups were briefed on the purpose and procedure of the experiment before written informed consent was obtained. They also provided written informed consent for publication. These experiments were conducted in a local Malaysia oil palm plantation. They were carried out in accordance with relevant guidelines and regulations and were approved by Monash University Human Research Ethics Committee (MUHREC)—Project number 18845. Once the IMUs and sEMG were placed on the participant’s body, the participant was requested to remain stationary in an upright posture for three seconds for sensor calibration purposes. The subjects then performed the required activity three times for 60 s with a 30 s break between trials. The activities were recorded throughout the experiment using a camera.

### Data processing

The EMG signals were processed in MATLAB (Mathworks, Nantucket, MA, USA). The signals were filtered using a band-pass filter with a cut-off frequency of 20 Hz and 450 Hz, followed by full-wave rectification and smoothing using a moving average filter with a window size of 100 ms^25, 33–35^. Due to the remoteness of the plantation, it was not feasible to adopt the conventional Maximum Voluntary Contraction (MVC) method to normalize the EMG signal. Instead, the Peak Dynamic Method (PDM)^36^ was used in this study. This method is known to be able to produce comparable and reliable results^36^. It uses the peak value of the muscle among the trials to normalize the EMG data, as defined in Eq. (1).

The regions of interest which correspond to FFB harvesting and LF collection were then identified. The recorded video was used to validate the time period when the activity occurred during the experiment. The normalized mean^27^ and peak of the EMG signal of each muscle during these harvesting periods were then determined, as shown in Eqs. (1) and (2), respectively.

1 $$EMG_{norm} \left( {mean} \right) = \frac{{EMG_{mean} }}{{EMG_{max} }} \times 100\%$$

where EMG_norm_(mean) = normalized mean EMG (%), EMG_mean_ = mean EMG during each harvesting period (mV), EMG_max_ = maximum EMG found among all trials (mV).

2 $$EMG_{norm} \left( {peak} \right) = \frac{{EMG_{peak} }}{{EMG_{max} }} \times 100\%$$

where EMG_norm_(peak) = normalized peak EMG (%), EMG_peak_ = peak EMG during each harvesting period (mV), EMG_max_ = maximum EMG found among all trials (mV).

The joint angles measured by the IMUs were filtered using 4th order Butterworth low pass filter with a cut-off frequency of 10-Hz and then smoothened by a moving average filter with a window size of 100 ms. The maximum joint angle was identified after eliminating the zero error. The normative ROMs of each joint were separated into five levels for the measurement of perceived discomforts: 0% (neutral), 25%, 50%, 75% and 100%^37, 38^. Each joint motion has a different discomfort value (DV) at a different level, indicating different quality of posture. DV value equal or greater than 63.5 indicates 'poor' posture, which has higher risk of MSD. DV value between 15.2 and 63.5 indicates 'so-so' postures, whereas DV value equal or less than 15.2 indicates good posture with low risk of MSD. For example, the measured maximum back joint extension was 18.23 degree, which corresponds to 72.92% of the normative ROM (25 degrees). This equals to the DV value of 48, indicating so-so quality of posture. The joint motions were then compared with the EMG results to confirm muscle behaviour during FFB harvesting and LF collection.

## Result and analysis

### Fresh fruit bunch harvesting

**Figure 4:**
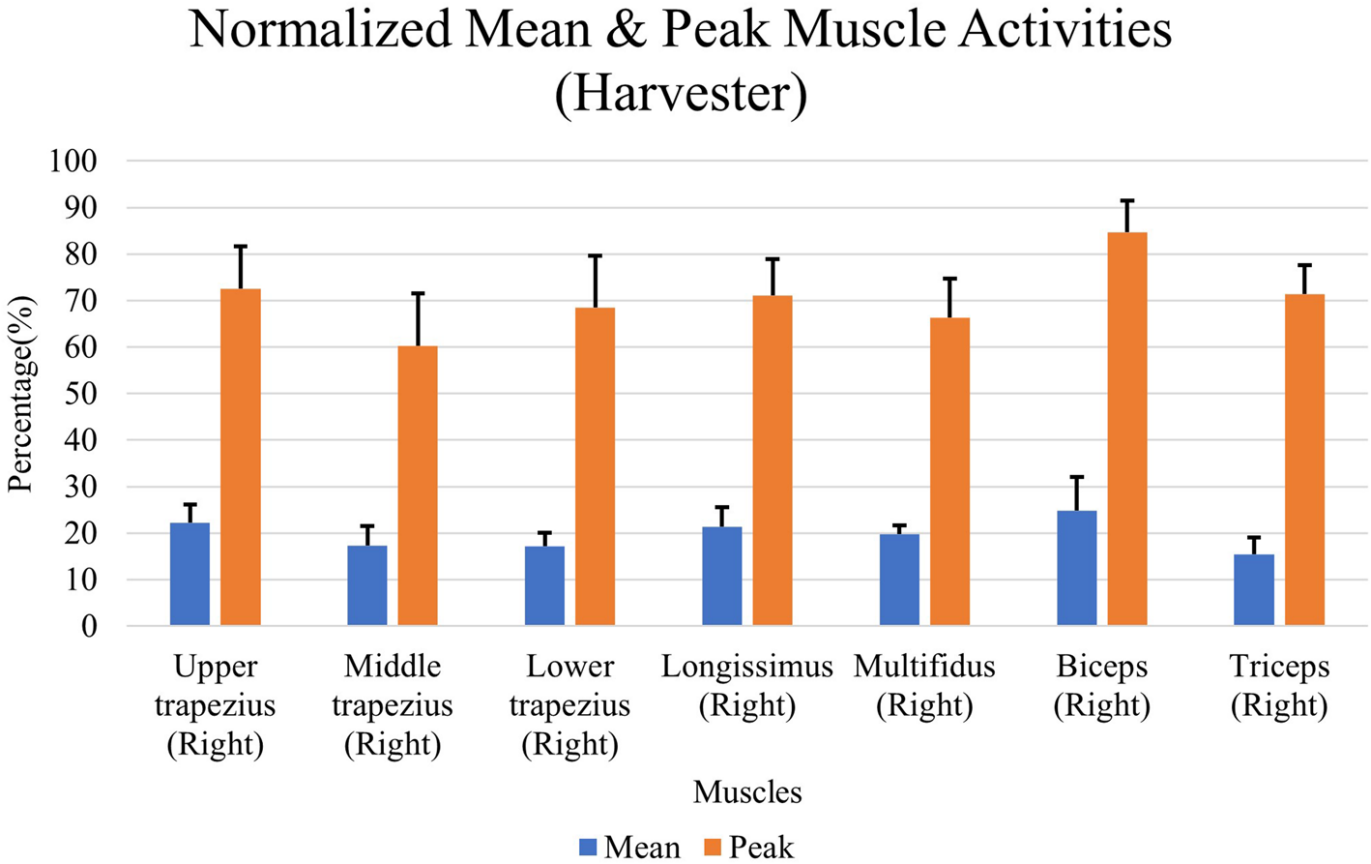
The average mean and peak muscle activities during FFB Harvesting.

The average mean and peak EMG activity during harvesting are presented in Fig. 4. It can be observed that the right biceps is the most active muscle—having the highest mean and peak values. Variations were found in the average activities of the remaining muscles. The right upper trapezius, right longissimus and right multifidus exhibited greater mean muscle activities ranging between 25 and 30%. The highest peak muscle activities (between 60 and 70%) were found in upper trapezius, lower trapezius, longissimus, multifidus, triceps. The triceps, middle trapezius and lower trapezius had relatively lower mean muscle activity than the others. The middle trapezius had the lowest normalized peak muscles activity, which indicates that it was less active than the other muscles investigated in this study.

**Figure 5:**
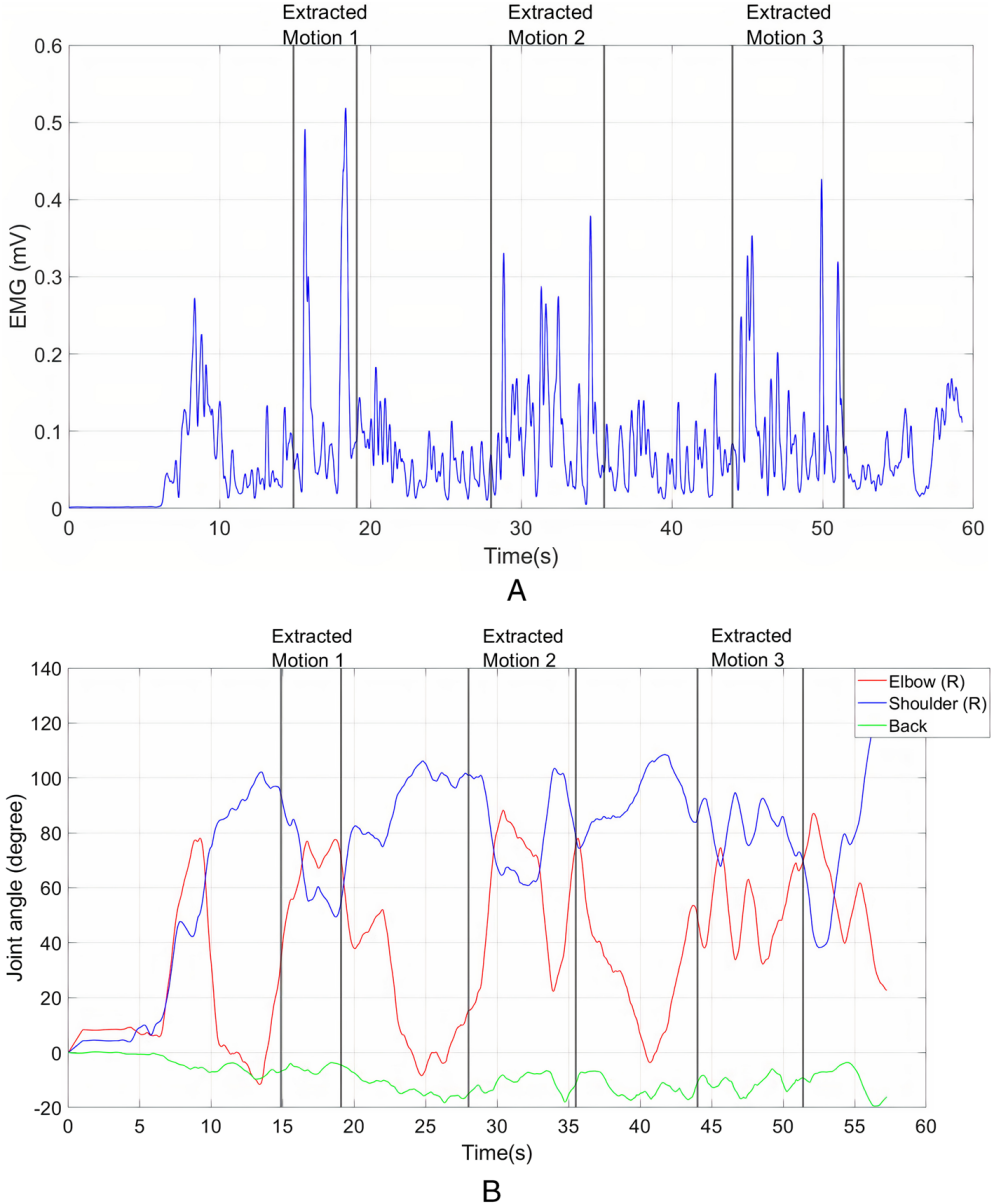
(**a**) EMG of right biceps muscle; (**b**) Right elbow, shoulder and back flexion and extension during FFB harvesting.

Figure 5a illustrates the EMG behaviour of the harvester's biceps muscle during harvesting. Meanwhile, Fig. 5b illustrates the joint angles (right elbow, right shoulder and back) behaviour during FFB harvesting. Significant peaks can be found in biceps EMG when the harvester performed the activity. Each peak corresponds to the harvester's attempt to cut the stalk using the sickle to harvest the FFB. No noticeable feature can be found in the kinematic behaviour of the elbow, shoulder and back.

The DV values for the back, shoulder and elbow were calculated to identify the quality of the harvesting posture. The percentage of FFB harvesters (n = 8) with different quality of posture for the back, shoulder and elbow were compiled and presented in Table 2. It was observed that 41.27% of the harvesters recorded a poor back extension posture, suggesting strenuous joint motion. Majority of the harvesters recorded a so-so posture for back lateral bending (73.02%), left shoulder internal rotation (58.73%), left shoulder flexion (66.67%), right shoulder flexion (71.43%), left elbow supination (76.19%), left elbow flexion (73.02%) and right elbow flexion (82.54%), indicating the potential of strenuous joint motions.

**Table 2:** Percentage of FFB harvesters with poor, so-so and good posture for different types of back, shoulder and elbow joint motion (n = 8).

Body part	Motion	Poor	So-so	Good
Back	Lateral bending	25.40%	73.01%	1.59%
	Rotation	0.00%	39.68%	60.32%
	Flexion	0.00%	15.87%	84.13%
	Extension	41.27%	39.68%	19.05%
Shoulder	Abduction (L)	0.00%	23.81%	76.19%
	Abduction (R)	0.00%	12.70%	87.30%
	Adduction (L)	0.00%	11.11%	88.89%
	Adduction (R)	0.00%	28.57%	71.43%
	Internal rotation (L)	0.00%	58.73%	41.27%
	Internal rotation (R)	0.00%	19.05%	80.95%
	External rotation (L)	0.00%	3.17%	96.83%
	External rotation (R)	0.00%	25.40%	74.60%
	Flexion (L)	0.00%	66.67%	33.33%
	Flexion (R)	0.00%	71.43%	28.57%
	Extension (L)	0.00%	9.52%	90.48%
	Extension (R)	0.00%	1.59%	98.41%
Elbow	Supination (L)	0.00%	76.19%	23.81%
	Supination (R)	0.00%	33.33%	66.67%
	Pronation (L)	0.00%	12.70%	87.30%
	Pronation (R)	0.00%	15.87%	84.13%
	Flexion (L)	0.00%	73.02%	26.98%
	Flexion (R)	0.00%	82.54%	17.46%

The relationship between muscle activities and joint kinematics are presented in Table 3. The contribution of each muscle to FFB harvesting activity could be observed from the normalized mean EMG and normalized peak EMG. It was found that both lower back muscles (longissimus and multifidus) which are responsible for back extension and back lateral bending, had high mean and peak EMG value. Moreover, among the motion investigated, around 25.4% to 41.27% of the harvesters shown "Poor" posture quality in lower back motion (back extension and back lateral bending). Hence, it can be deduced these harvesters have a high probability of facing lower back MSD. Biceps, responsible for shoulder flexion, elbow supination and elbow flexion, is another critical muscle used during FFB harvesting. It has the highest normalized mean and peak activation among the muscles investigated. Most of the elbow and shoulder range of motion of the harvesters investigated were categorized as a "So-so" posture. In other words, the probability of the occurrence of elbow and shoulder MSD is lower than the lower back but may still occur. Hence, the posture quality must still be improvised to minimize the likelihood of getting MSD. Other muscles, such as the upper, middle and lower trapezius, were not included here as the IMUs did not measure the scapulocostal joint. Only the kinematics behaviour of the back, shoulder and elbow joints were examined in this work.

**Table 3:** A relationship between the potential stressful joint motions with their associated muscles.

Joint motions	Muscles
Back extension	Longissimus Multifidus
Back lateral bending	Longissimus Multifidus
Shoulder flexion	Biceps
Elbow supination	Biceps
Elbow flexion	Biceps

### Loose fruit collection

**Figure 6:**
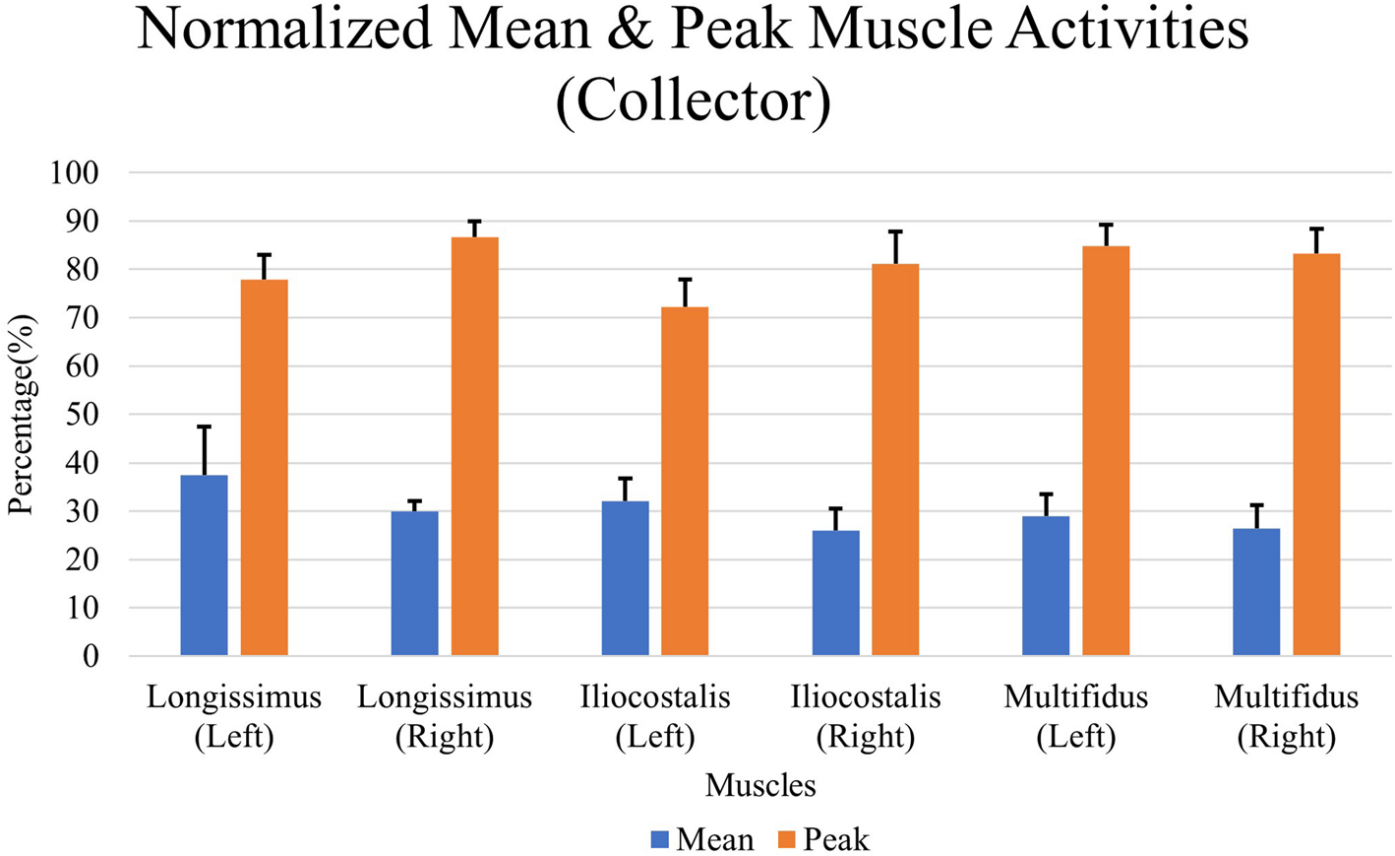
The average mean and peak EMG activities during LF collection.

The average mean and peak EMG activities of the muscles during LF collection is presented in Fig. 6. It can be observed that the first three muscles, namely left longissimus, left iliocostalis and right longissimus have the largest average muscle activities, ranging between 30 and 40%. The remaining muscles had an average mean value below 30%. On the other hand, the right longissimus, right multifidus, and left multifidus produced the largest peak EMG signal , ranging between 80 and 90%.

**Figure 7:**
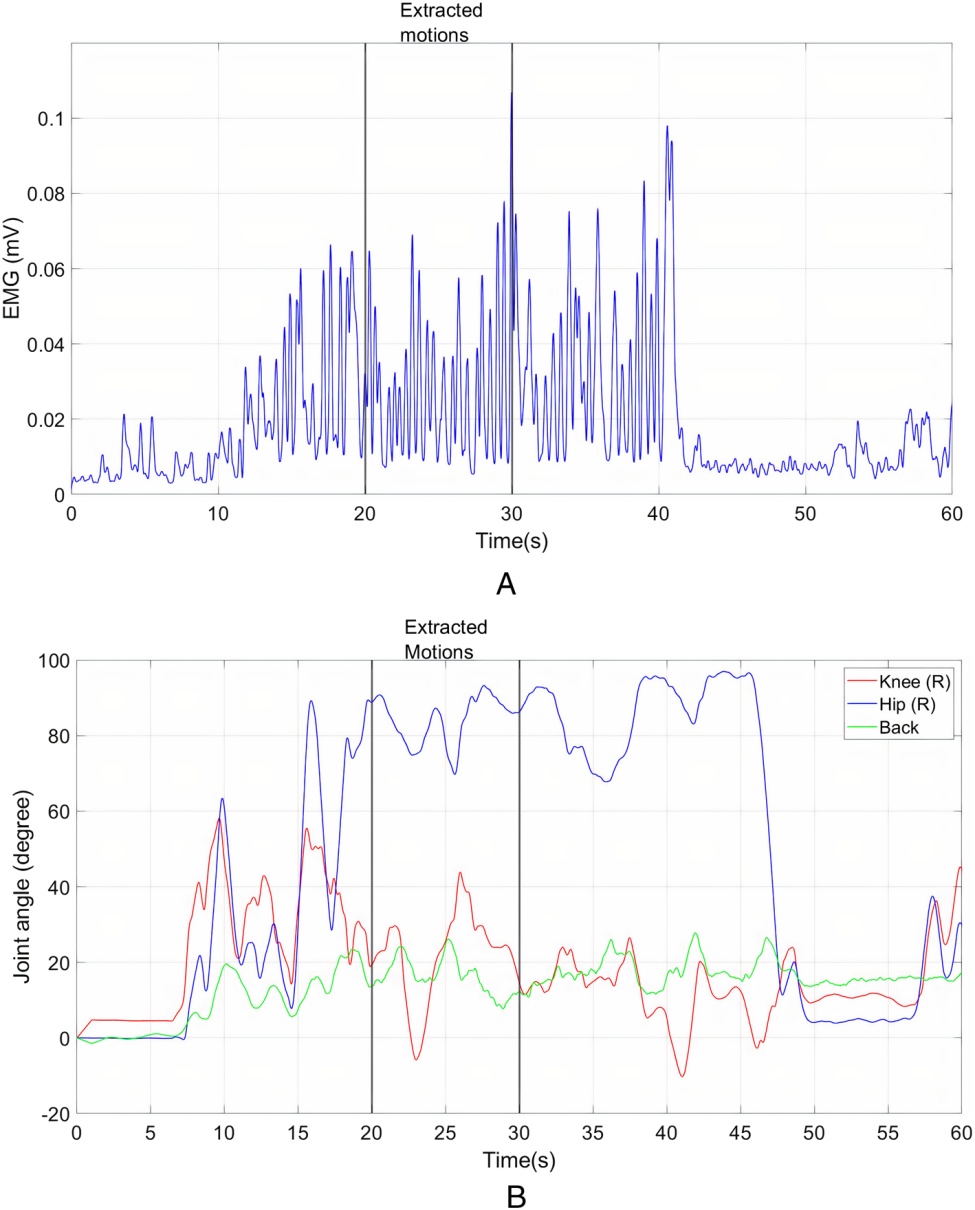
(**a**) EMG of right longissimus muscle (**b**) Right knee, right hip and back flexion–extension during LF collection.

Figure 7 shows the activation of the right longissimus muscle and kinematics of the right knee and right hip during LF collection. It can be seen that the collector had to flex his back, slightly bend his knee, and maintain this posture to collect the loose fruits from the ground. Minor knee extension was observed at approximately 22 s after the start of the experiment. This motion was performed by the collector to conform to the uneven surface of the ground surrounding the oil palm tree.

The DV values for the back, hip and knee were calculated to identify the quality of the posture. The percentage of LF collectors (n = 8) with different quality of posture for the back, hip and knee were tabulated in Table 4. It was found that most collectors demonstrated poor postures at the left hip external rotation (66.67%), left hip flexion (100.00%) and right hip flexion (100.00%), suggesting strenuous joint motions. So-so postures were observed at the back lateral bending (76.19%), back rotation (80.95%), back flexion (76.19%), left hip abduction (52.38%), right hip abduction (61.90%), left hip adduction (95.24%), right hip adduction (100.00%), left hip internal rotation (76.19%), right hip internal rotation (61.90%), right hip external rotation (76.19%), left knee flexion (90.48%) and right knee flexion (95.24%).

**Table 4:** Percentage of LF collectors with poor, so-so and good posture for different types of back, hip and knee joint motion (n = 8).

Body part	Motion	Poor	So-so	Good
Back	Lateral bending	23.81%	76.19%	0.00%
	Rotation	0.00%	80.95%	19.05%
	Flexion	0.00%	76.19%	23.81%
	Extension	14.29%	38.10%	47.62%
Hip	Abduction (L)	19.05%	52.38%	28.57%
	Abduction (R)	23.81%	61.90%	14.29%
	Abduction (L)	0.00%	95.24%	4.76%
	Abduction (R)	0.00%	100.00%	0.00%
	Internal rotation (L)	23.81%	76.19%	0.00%
	Internal rotation (R)	38.10%	61.90%	0.00%
	External rotation (L)	66.67%	33.33%	0.00%
	External rotation (R)	23.81%	76.19%	0.00%
	Flexion (L)	100.00%	0.00%	0.00%
	Flexion (R)	100.00%	0.00%	0.00%
	Extension (L)	19.05%	4.76%	76.19%
	Extension (R)	9.52%	4.76%	85.71%
Knee	Flexion (L)	0.00%	90.48%	9.52%
	Flexion (R)	0.00%	95.24%	4.76%

The relationship between muscle activities and joint kinematics are presented in Table 5. The normalized mean and peak EMG for all the muscles are listed. The LF collection heavily relied on the back, and lower extremity muscles as the collectors had to bend their back to collect the scattered loose fruits on the ground. This behaviour is reflected in the muscles activations of the back muscles such as iliocostalis, longissimus and multifidus, and the kinematics of the hip and knee. 10% to 20% of the collectors had a bad posture during LF collecting, indicating a high probability of getting MSD (Table 4). The results suggest that hips is the body part which MSD is most likely to occur in LF collectors. All the eight collectors exhibited "Poor" posture at the left and right hip flexion during the activity. The muscles responsible for the hip and knee motion were not investigated here, as it requires the collectors to be in short tights in a public area (plantation) while performing their duties. Doing so exposes the collectors to the potential harms caused by mosquitoes, leeches, and other insects. Nevertheless, despite the lack of measurement of these muscle groups, it can be confirmed that all the back muscles (longissimus, iliocostalis, multifidus) are the primary muscles used during LF collection based on their significant muscle activations and their corresponding strenuous joint motions.

**Table 5:** A relationship between the potential stressful joint motions with their associated muscles.

Joint motions	Muscles (Rank_mean,left_, Rank_mean,right_, Rank_peak,left_, Rank_peak,right_)
Hip external rotation	–
Hip flexion	–
Back lateral bending	Iliocostalis Multifidus Longissimus
Back rotation	Iliocostalis Multifidus Longissimus
Back flexion	–
Hip abduction	–
Hip adduction	–
Hip internal rotation	–
Hip external rotation	–
Knee flexion	–

## Discussions

This study presents a kinesiological and kinematic test of the FFB harvesters and LF collectors using direct measurements, evaluating different joint motions and muscle activation (reliance). It was found that the back extension, back lateral bending, shoulder flexion, elbow supination and elbow flexion were the potential strenuous joint motions during the FFB harvesting. Biceps, upper trapezius, longissimus and multifidus were found to be the muscles that are heavily used.

Numerous studies with qualitative approaches and without any direct measurements such as survey and observation have shown that oil palm harvesters, in general, suffer from lower back pain^2, 7, 39, 40^. In their recent study, *Sirothorn Tewtow *et al*.* investigated the prevalence of MSD among oil palm workers in Thailand. They found that 71.2% of harvesters complained of discomfort in the lower back, followed by neck (63.5%), shoulder (59.6%), elbow (40.4%) and hand (40.4%)^41^. Other studies, such as^2, 7, 39^ and a systematic review of occupational hazards among the oil palm plantation workers by Nuruly Myzabella et al*.*^40^. revealed a similar trend: the workers experienced lower back pain. These studies agree well with our findings. The workers were found to rely heavily on their lower back muscles. During harvesting, they actively used longissimus and multifidus muscles to harvest the FFB.

The results presented in this study are also consistent with the study by Faiz Syuaib^42^, who investigated the ergonomic risks associated with harvesting tasks by male harvesters in Indonesia. Their study analyzed the anthropometric dimensions, work motion and posture of the harvesters relying on the video recording of the harvesting motions. They found that the joint motions that posed a risk of hyperflexion are the shoulder and elbow, whereas joint motion that posed a threat of hyperextension is the back. These motions essentially concur with our findings of potential strenuous joint motions. Additionally, it was also reported that the neck extension was also the joint motion at a high risk of injury—this too was consistent with our finding where we found the upper trapezius muscle to have a significant muscle activity^30, 42^.

During LF collection, the hip external rotation, hip flexion, back lateral bending, back rotation, back flexion, hip abduction, hip adduction, hip internal rotation, hip external rotation and knee flexion were observed to be the potential strenuous joint motions. Additionally, the right longissimus and iliocostalis were the muscles, which showed high activation during this activity, indicating a high prevalence of MSD on the lower back. These findings are in agreement with a previous study by Nur Syazwani et al., where the ergonomic risk of LF collectors was investigated. The previous study reported that the lower back (36.4%) showed the highest prevalence of MSD, followed by a calf (33%), buttock (28.4%) and left knee (28.4%)^7^. The buttocks which are made up of three muscles in the gluteal group are responsible for all the movements of the hip joint. Hence it is consistent with our findings ^30^. Our results are also found to be compatible with a recent study, which reported that the lower back (88.4%) of collectors showed the highest prevalence of MSD, followed by the knee (60.5%) and hip (46.5%)^41^.

In other related areas, a few reports investigated the prevalence of MSD of solid waste collectors, who perform similar motions as LF collectors. Pamela Castro et al. found out that solid waste collectors show discomfort on the dorsal spine, lumbar and knees through a survey. The authors verified the result by using a thermographic image, confirming the back region showed the highest risk of MSD^43^. As pointed out by Mostafizur Rahman, 36.9% and 41.8% of solid waste collectors complained of suffering back pain and joint pain, respectively. These findings correlate well with other studies. 91.3% of collectors in Malaysia faced lower back pain, whereas 41.8% of collectors in Dhaka faced pain in the joint and 36.9% faced pain in the back^44^. These results suggest that the back, hip and knees are the potential prevalence of MSD for LF collectors and other individuals with a similar posture requirement in different fields of job.

Overall, this quantitative study concurs with previous qualitative studies for both FFB harvesting and LF collection, confirming the high prevalence of MSD on various parts of the body among harvesters and collectors, mostly lower back. In addition, this study further provides essential information on the harvesters and collectors in terms of muscle activations and joint kinematics. This information is beneficial for further research on investigating deep muscles which cannot be measured by sEMG but might contribute significantly to both FFB harvesting and LF collection. These deep muscles, alongside the missing muscles and joints due to the limitation of sensors and/or ethical issues are recommended to be estimated by using musculoskeletal software such as OpenSim. By importing the kinematic data obtained in this study, the motions of FFB harvesting and LF collection could be simulated to get the muscle activations and joint kinematics. The validation of the reliability should be supported by the comparison of the experimental and the simulated EMG activations^45^. Moreover, due to confirmed results via direct measurements, a wearable robot prototype which could provide force support^46^ to the harvesters and collectors could be developed based on the EMG activations and motion analysis obtained in this study^47^.

## Conclusion

To the best of our knowledge, the described work is the first study which applied a quantitative approach via direct measurements, by using EMG and IMUs sensors, on oil palm FFB harvesters and LF collectors. Our findings support the previous qualitative studies that the harvesters and collectors are suffering the high ergonomic risk of MSD on various parts of the body mostly the lower back. Moreover, we have proved that the biceps are another important muscle commonly relied on by the harvesters during FFB harvesting. Due to confirmed results via direct measurements, this study is precious for further research such as the development of a musculoskeletal model and assistive devices like a wearable robot which could reduce the risk of MSD of both FFB harvesters and LF collectors.
